# Open reduction internal fixation vs non-operative management in proximal humerus fractures: a prospective, randomized controlled trial protocol

**DOI:** 10.1186/s12891-018-2223-3

**Published:** 2018-08-18

**Authors:** Lisa Howard, Randa Berdusco, Franco Momoli, J. Pollock, Allan Liew, Steve Papp, Karl-Andre Lalonde, Wade Gofton, Sara Ruggiero, Peter Lapner

**Affiliations:** 1 Division of Orthopaedic Surgery, Ottawa Hospital Research Institute, University of Ottawa, Ottawa, ON Canada; 2 Orthopaedic Sports Medicine & Upper Extremity Reconstruction, Fellowship, University of Manitoba, Winnipeg, MB Canada; 3 Ottawa Hospital Research Institute, Ottawa, Canada; 4 School of Epidemiology, Public Health, and Preventive Medicine, University of Ottawa, Ottawa, Canada; 5 Children’s Hospital of Eastern Ontario Research Institute, Ottawa, Canada

**Keywords:** Shoulder, Shoulder joint, Open reduction internal fixation (ORIF), Locked plating, Randomized controlled trial, Proximal humeral fracture

## Abstract

**Background:**

Proximal humerus fractures are the third most common fracture in the elderly population and are expected to increase due to the aging population. Surgical fixation with locking plate technology has increased over the last decade despite a lack of proven superiority in the literature. Three previous randomized controlled trials have not shown a difference in patient-centered outcomes when comparing non-operative treatment with open reduction and internal fixation. Low patient enrollment and other methodological concerns however limit the generalizability of these conclusions and as a result, management of these fractures remains a controversy. By comparing the functional outcomes of locked plate surgical fixation versus non-operative treatment of displaced three and four-part proximal humerus fractures in the elderly population with a large scale, prospective, multi-centered randomized controlled trial, the optimal management strategy for this common injury may be determined.

**Methods:**

We will conduct a prospective, single blind randomized controlled parallel arm trial to compare non-operative management of proximal humerus fractures with open reduction and internal fixation using locked plating technology. One-hundred and sixty patients > age 60 with acute 3- or 4- part proximal humerus fractures will be randomized to either open reduction and internal fixation with locked plating technology or non-operative management treatment arms. The primary outcome measure is the Constant Score at 24 months post-operative. Secondary outcome measures include the American Shoulder and Elbow Surgeon’s Score (ASES), EuroQol EQ-5D-5 L Health Questionnaire Score, short form PROMIS upper extremity score and IPAQ for the elderly score. Further outcome measures include assessment of the initial classification, displacement and angulation and the quality of surgical reduction via a standard computed tomography (CT) scan; rates of non-union, malunion, arthrosis, osteopenia or other complications including infection, nerve injury, intra-articular screw penetration, reoperation rates and hospital re-admission rates.

**Discussion:**

The results of this trial will provide Level 1 evidence to guide decision-making in the treatment of proximal humerus fractures in the elderly population.

**Trial registration:**

ClinicalTrials.gov NCT02362100. Registered 5 Feb 2015.

## Background

Proximal humerus fractures account for 6% of all fractures [1] and are the third most common extremity fracture in the elderly population following hip and distal radius fractures [2]. Associated with an aging population, the incidence of osteoporotic proximal humerus fractures is increasing and is expected to triple over the next three decades [3]. The majority of proximal humerus fractures are minimally displaced and can be treated non-operatively. Controversy remains regarding the optimal care of displaced fractures with potential treatment options of non-operative management, percutaneous fixation, open reduction internal fixation and arthroplasty [4–8].

### Non-operative treatment of displaced fractures

Non-operative management of proximal humerus fractures with a period of immobilization and progressive physiotherapy is a simple, noninvasive and readily available treatment option. In a systematic review of non-operative management, Iyengar et al. [9] evaluated 12 studies (*n* = 650) [10–21], with a mean age of 65.0 years and a mean follow-up of 3.8 years (range of 1–10 years). Based on the Neer classification [22], there were 49% undisplaced or one-part (*n* = 317), 25% two-part (*n* = 165), 21% three-part (*n* = 137), and 5% four-part (*n* = 31) fractures. Although variable, all treatment protocols included a period of sling immobilization followed by progressive mobilization as tolerated. The mean rate of radiographic union was 98% (range 93–100%). Various functional outcome scores were used; with 6 studies (*n* = 272) [10, 12, 14, 19–21] showing a weighted mean Constant score of 74 (range 55–81) corresponding to a “fair” outcome. Across all studies, a 13% complication rate was reported, with varus malunion being the most common (*n* = 44 or 7%). Proximal humerus avascular necrosis was found to be uncommon (*n* = 13 or 2%) [9]. In the largest included trial, Hanson et al. reported the functional outcomes of non-operative management through a prospective evaluation of 160 patients, with 124 patients having complete 1-year follow-up. Nearly half (53.1%) were undisplaced fractures. The average Constant score was 74.3 with a mean difference between the injured and contralateral shoulder of 8.2. They found an estimated median time to definitive union of 14 weeks, and a 7% risk of delayed or nonunion. Four patients went on to require surgical fixation and 5 patients underwent arthroscopic decompression, with an eventual operation rate of 5.6% [12]. With a large focus on undisplaced fractures, these studies highlight that non-operative management of proximal humerus fractures can lead to satisfactory functional outcomes with modest complication rates. In a report of non-operative management of displaced proximal humerus fractures, Yuksel et al. reported a mean Constant score of 61.3 (*n* = 18, eight 3-part and ten 4-part; mean age of 68.2 years; mean follow-up of 3.3 years), with nonunion and osteonecrosis detected in 27.8% (*n* = 5) [23].

### Surgical fixation of displaced fractures

Displaced proximal humerus fractures are commonly treated with open locking plate fixation [4–6, 8, 24–26]. A systematic review of 514 displaced proximal humerus fractures treated with locking plate fixation (12 included studies, average age of 62, average follow-up of 2.4 years) [27–38] showed an overall healing rate of 96.6%. The review included 34.0% two-part (*n* = 175), 44.7% three-part (*n* = 230), and 21.2% four-part (*n* = 109) fractures. Nine out of the 12 studies (*n* = 376) [27–29, 32, 33, 35–38] reported an average Constant score of 73.6 when evaluating functional outcome. When stratified for fracture classification, the Constant score was significantly less for 4-part fractures in comparison to the 2-part fractures (*p* = 0.02). The overall complication rate was 48.8% with a reoperation rate of 13.8%. With the exclusion of varus malunion, the complication rate remained high at 32.6% over the 12 studies analyzed [24]. Two other multicenter studies evaluated locking plate fixation for the treatment of displaced proximal humerus fractures reported similar Constant scores of 70.6 and 72 at a minimum of 1 year follow-up, and overall complication rates of 40 and 45% [25, 26]. The Proximal Fracture of the Humerus Evaluation by Randomisation (PROFHER) trial was another multi-centered randomized controlled trial of 250 patients > age 16 which showed no difference between operative and nonoperative management using the Oxford shoulder score and the Short Form 12 (SF-12). The complications in the surgical and nonsurgical group were reported as 24% and 18% respectively [39].

Despite the lack of superiority demonstrated, there has been a significant increase in surgical fixation of proximal humerus fractures following the introduction of locking plate technology over the last decade [40, 41]. Associated with high complication rates, open reduction and internal fixation of isolated proximal humerus fractures in the elderly has also been found to be an independent risk factor for inpatient adverse events and mortality [42].

Previous comparisons of non-operative and locking plate fixation have been conducted with no clear consensus as to the optimal management of osteoporotic proximal humerus fractures. No difference in the quality adjusted life years (QALYs) and societal costs between the two treatment options have been demonstrated [43]. In a randomized control trial evaluating 60 displaced 3-part proximal humerus fractures, Olerud et al. found no statistically significant difference in functional outcomes (Constant, Disability of the Arm Shoulder and Hand (DASH), and health-related quality of life (HRQoL) scores) between non-operative management and locking plate fixation over a 2-year period. One patient (3%) in the non-operative group went on to require surgical intervention and 9 patients (30%) in the locking plate group had a secondary operation with a major complication rate of 13% [44]. Fjalestad and Hole have recently reported on 50 elderly patients with displaced proximal humerus fractures randomized to non-operative or operative management using locking plate fixation. Fracture patterns were categorized based on the AO/OTA classification system, which makes a direct comparison to the study by Olerud et al. difficult. With the Constant score as their primary outcome, Fjalestad and Hole found no significant functional or HRQoL difference over a 2-year follow-up period. Similar to previous studies [25, 26], a 35% overall complication rate with surgical management was reported [45]. Both randomized control trials were limited by a small number of enrolled patients [44, 45].

It is generally accepted that non-operative management is ideal for undisplaced proximal humerus fractures, while displaced four-part fractures can be treated with non-operative management, surgical fixation or arthroplasty options [5, 6]. With no consensus in the literature, the specific management of displaced two and three-part proximal humerus fractures remains highly variable, with non-operative and locking plate surgical fixation the two most common and readily available treatment options. Given the lack of consensus on optimal treatment, conflicting, low quality-of-evidence reports, and higher level of evidence studies beset by various limitations, treatment remains highly controversial. By comparing the functional outcomes of surgical fixation versus non-operative treatment of displaced two, three and four-part proximal humerus fractures in the elderly population, the optimal management strategy for this common injury may be determined. The results of this trial would have the potential to minimize unnecessary complications and provide much needed guidance to orthopedic surgeons striving to maximize patient function and provide quality patient care in an era of rapidly increasing health care costs.

## Objectives

### Primary objective

A. Our primary objective is to determine if there is a difference in the functional outcome between non-operative management and locking plate surgical fixation of low-energy displaced three- and four-part proximal humerus fractures in the elderly population based on the Constant functional outcome score [46] over a 2-year follow-up period.

### Secondary objectives

A. Is there a difference between non-operative management and locking plate surgical fixation of low-energy displaced three- and four-part proximal humerus fractures in the elderly population based on the ASES functional outcome score [47], the short form Patient Reported Outcomes Measurement Information System (PROMIS) upper extremity score [48], the International Physical Activity Questionnaire (IPAQ) for the elderly [49], and the EuroQol EQ-5D-5 L Health Questionnaire Quality of Life (QoL) functional outcome score [50] over a 2-year follow-up period? What is the incidence of complications of non-operative management and locking plate surgical fixation of low-energy displaced three- and four-part proximal humerus fractures in the elderly population based on infection, nerve injury, intra-articular screw penetration and bleeding (hematoma), reoperation rate, or hospital readmission over a 2-year follow-up period
B. Does a difference exist between non-operative management and locking plate surgical fixation of low-energy displaced three- and four-part proximal humerus fractures in the elderly population based on radiographic outcomes including time to union, non-union, malunion, and joint arthrosis?
C. Does the degree of initial displacement or angulation of the fracture fragments correlate with final functional outcome measures?
D. Does the quality of the surgical reduction correlate with final functional outcome measures?

## Methods

### Study design

This study is designed as a single-centered prospective, single blind, randomized controlled trial (RCT) with parallel arms comparing nonoperative management with locked plate open reduction and internal fixation (ORIF) of proximal humerus fractures. After enrollment, patients will be randomly allocated to receive either nonoperative or operative fixation of their proximal humerus fractures. This study will abide by the current international research standards and will be reported as per the guidelines in the CONSORT statement [51]. Approval was obtained from the Health Science Network Research Ethics Board of Ottawa and is in compliance with: Ethical Conduct for Research Involving Humans; the International Conference Harmonization Good Clinical Practice: Consolidated Guideline and the provisions of the Personal Health Information Protection Act 2004. All surgical procedures will be performed by fellowship trained shoulder surgeons in a large University-affiliated hospital.

### Purpose and hypothesis

The main purpose of this trial is to determine whether or not a functional difference exists between operative and nonoperative management of low-energy displaced three- and four-part proximal humerus fractures as measured by the Constant, (PROMIS) upper extremity score, the International Physical Activity Questionnaire (IPAQ) for the elderly, and the EuroQol EQ-5D-5 L Health Questionnaire Quality of Life (QoL) functional outcome score [46, 48–50] over a period of 24 months. We will also aim to determine which method of treatment is associated with a higher incidence of complications as well as the time to union of malunion, nonunion and joint arthrosis. Lastly, we will determine whether initial displacement or quality of surgical reduction has an impact on functional outcome.

We hypothesize that there will be no statistically significant difference in functional outcomes between operative and nonoperative methods. We also hypothesize that there will be a greater incidence of complications in the operative group. Lastly, we hypothesize that fractures with a greater Neer classification and those with a greater degree of initial displacement will have worse functional outcomes.

### Participants

Patients will be screened in the emergency department of a large University-affiliated hospital and will be enrolled in the fracture clinic. Table 1 lists eligibility criteria for the study. Enrolled patients include males and females > age 60 with acute (< 3 weeks) displaced proximal humerus fractures that fall into the Neer category of 3- or 4- part. Diagnosis will be obtained from radiographs including a true AP (neutral rotation) of the shoulder, a lateral Y-view and an axillary (or trauma axillary) view. The fractures reviewed for inclusion will be independently assessed by two Orthopaedic Surgeons participating in the trial. If there is any disagreement on the classification or inclusion, a third surgeon will be asked to review. The majority consensus will be the implemented inclusion and classification. Eligible patients that have consented to participate in the study will receive informed consent on the two treatment arms prior to randomization. Preoperative baseline functional assessment including the Constant, ASES, EQ-5D-5 L, PROMIS and IPAQ scores will be completed. Patients randomized to the surgical arm will undergo surgery within 7 days of presentation.

**Table 1:** Inclusion and Exclusion Criteria

Inclusion Criteria	
1. Displaced 3-part proximal humerus fractures by the Neer classification; or displaced 4-part proximal humeral fractures by the Neer classification that are deemed amenable to surgical internal fixation	
2. > 60 years of age	
3. Low energy mechanism of injury	
4. Acute fracture^a^	
Exclusion Criteria	
1. 4-part proximal humerus fractures that are not deemed amenable to surgical fixation^b^; fractures that are better suited to treatment with arthroplasty	
2. Isolated greater tuberosity fractures	
3. Ipsilateral upper extremity significant injury, concomitant fracture or polytrauma	
4. Open fracture	
5. Previous ipsilateral shoulder surgery	
6. Patients with active worker’s compensation claims^c^	
7. Active joint or systemic infection	
8. Patients with convulsive disorders, collagen diseases, and any other conditions that might affect the mobility of the shoulder joint	
9. Major medical illness^d^	
10. Unable to speak or read English/French	
11. Psychiatric illness that precludes informed consent	
12. Unwilling to be followed for 2 years	

^a^ <3 weeks

^b^due to osteopenic bone, thin head or tuberosity fragments

^c^due to the expectation of lower rates of success in this patient population

^d^life expectancy less than 2 years, unacceptably high operative risk, or not medically cleared by preoperative anesthesia consult

### Sample size calculation

The sample size will be 160 patients. The minimum clinically important difference for the Constant score is 12 [52, 53], with standard deviation of 23.1 from measurements in previous studies of similar patient populations. For the primary outcome, to achieve 80% power to detect a clinically meaningful difference of 12 points on the Constant score, with standard deviation of 23.1, and alpha of 0.05, a sample size of approximately 116 people would be necessary (58 per arm). The overall sample size is increased from 116 to 129 to account for an expected 5% crossover from the non-operative study arm to the operative study arm. An additional 31 patients were added for a conservative sample size adjustment accounting for 20% loss-to-follow-up over the two years of follow-up, for a total sample size of 160 patients.

### Randomization and blinding

**Fig. 1:**
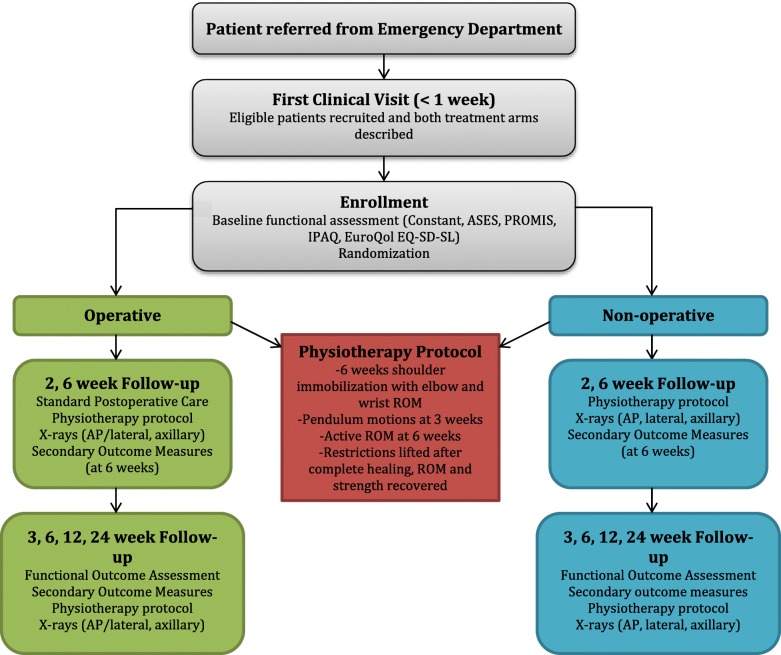
Flow of patients through the trial

Figure 1 shows the flow of procedures in the trial. Study group allocations will have been pre-determined from an online randomization generator and catalogued in sealed envelopes by personnel independent of the study. The allocation will be in a 1:1 ratio, stratified by fracture type (3 and 4 part): 3- and 4-part fractures will be stratified using permuted blocks of variable length (4 to 6). Randomization and allocation to treatment will be determined on the day of the first assessment in the plaster room clinic within 7 days post injury or at the time of the emergency room visit if the patient is unable to return home. The research coordinator will open the sealed envelope containing the study allocation and will inform the surgeon of the patient’s assigned treatment: non-operative or ORIF treatment. Patients assigned to the ORIF group will have surgery scheduled within 7 days following injury.

The research coordinator will enter all necessary patient information into a password protected electronic database, however due to the nature of the trial design, it is not possible for the surgeon (or the patient) to remain blinded to treatment allocation. The research coordinator will carry out the follow-up assessments and will remain blinded to the patient’s treatment allocation and will not have access to the participant’s chart or radiographs prior to or during the assessment. This will minimize the potential for biases introduced by the examiner when performing the physical assessment and recording data. To help reduce the potential for observer bias, the physical examination and the administration of study questionnaires are standardized. A trained musculoskeletal radiologist (AS) will perform the radiological assessments.

### Interventions

#### Non-operative treatment

Treatment will consist of sling immobilization for a period of 6 weeks. Patients will remain in a shoulder immobilization sling for 6 weeks with range of motion of the elbow, hand and wrist with addition of pendulum exercises from 3 weeks. Active mobilization will occur after 6 weeks. Light activity and range of motion in physiotherapy will be permitted at this time. Restrictions will be lifted and full function permitted once range of motion and strength have recovered.

#### Operative treatment

This trial will implement a standardized operative management protocol, using the Synthes TM (Synthes Canada, Mississauga, Ont., Canada) 3.5 mm LCP proximal humerus locking plate for open reduction internal fixation. Six fellowship-trained shoulder surgeons will perform the surgical fixation. Pre-operative medical clearance will be established via anesthesia consults if required for medically complex patients. Pre-operative intravenous (IV) antibiotic prophylaxis and administration of a general anesthetic will be utilized. Beach-chair position and the standard deltopectoral approach or minimally invasive plating technique will be utilized. The reduction technique will depend on the fracture pattern but key steps include disimpaction of the humeral head with anatomic reduction of the medial calcar and tuberosities. The locking plate will be positioned posterior to the bicipital groove at a target height of 5–8 mm distal to the tip of the greater tuberosity. Additional tuberosity suture fixation to the plate will be used when warranted. Provisional fixation, plate positioning and final fixation will be assessed with intraoperative fluoroscopy to ensure adequate reduction and hardware position. The surgical incision will be examined and staples removed 10–14 days postoperatively. The postoperative rehabilitation protocol will be identical to the nonoperative protocol as per above.

### Outcome measures

Outcome measures are separated into primary and secondary as outlines below. All functional assessment scores will be completed at baseline and 3, 6, 12 and 24 months post injury. Complications and adverse events will be recorded on standardized case-report forms that will be completed immediately following the completion of the surgical procedure for patients randomized to the surgical treatment arm. At follow-up visits, clinical evaluation will be conducted to monitor for complications including infection, nerve injury, and hematoma formation or hospital re-admission.

### Primary outcome measures

#### Constant score [46]

The Constant score has been validated and normalized in comparison to disease free patients and places greater emphasis on range of motion and strength and has been adopted by the European shoulder society for functional assessment of the shoulder. The Constant Score records a variety of shoulder measurements including an objective test of strength using a spring-loaded measuring device and reflects an overall clinical functional assessment. This instrument is based on a 100-point scale.

#### The American shoulder and elbow Surgeon’s (ASES) score [47]

The ASES score is a shoulder specific assessment tool developed by the American Shoulder and Elbow Society that consists of both patient self-assessment and physician assessment. It is a patient scoring system calculated from a self-assessment portion that evaluates pain and ability to perform tasks of daily living, and a clinical assessment which tests active range of shoulder motion and strength. The patient self-evaluation is divided into pain (recorded on a visual analogue scale) and activities of daily living (ADL, recorded on a numeric scale). The overall score is an equal weight of the two self-evaluation sections and produces a score out of 100 where 100 is the better outcome. The physician assessment is divided into four segments: range of motion, physical signs, strength and instability and does not provide a score.

#### The EuroQol EQ-5D-5 L health questionnaire score as a quality of life (QoL) measure [50]

It is a generic health status questionnaire, consisting of five dimensions (mobility, self-care, usual activities, pain/discomfort, anxiety/depression) each of which can take one of five responses. It also includes a visual analogue scale for recording an individual’s rating of their current health-related quality of life (scale 0 to 100).

#### The short form PROMIS upper extremity score [48]

This score asks 16 questions related specifically to the physical function that allows for a more precise assessment of the upper limbs. The questions ask whether the participant is able to do a collection of tasks that vary in terms of difficulty of the task, such as “are you able to peel fruit?” or “are you able to use a hammer to pound a nail?” There are five possible responses per question, ranging from ‘unable to do’ to ‘without any difficulty’.

#### The IPAQ for the elderly [49]

This is a short, four question survey geared towards the elderly population that asks respondents about the kinds of physical activities they do as part of their everyday lives. The IPAQ asks how much time in the last 7 days was spent sitting, walking, doing moderate intensity physical activity, and vigorous intensity physical activity.

### Secondary outcome measures

#### Classification, displacement, angulation and quality of reduction

Pre-operative plain films will include a true AP, axillary, and Y-lateral view. Post-injury radiographs including true AP, axillary views and y-lateral views will be obtained at the first post-operative visit (or 2 weeks post immobilization in the non-operative treatment arm), 6 weeks and 3, 6, 12 and 24 months following surgery. A standard computed tomography (CT) scan of the shoulder (pre operatively in all patients and immediately post-operatively in operative treated patients) will be conducted to ensure correct determination of the true nature of the fracture (3 or 4 part fracture as per the Neer [22], the OTA/AO [54] and the Hertel [55] classifications) as well as the degree of angulation and displacement of the fracture fragments. As per the Neer [22] classification, a “part” will be defined as displacement greater than 1 cm or angulation greater than 45 degrees. The OTA/AO classification [54] will also be determined on the pre-operative CT scans and classified as type A, B or C. The Hertel classification is based on a binary descriptive system that describes five basic fracture planes and twelve fracture patterns between the head, lesser tuberosity, greater tuberosity and the shaft components [55]. Pre-operative and post-operative (in the ORIF group) CT scans will be reformatted along standardized planes to determine fracture fragment position and angulation. The neck- shaft angle will be defined as the angle between the proximal humeral metadiaphysis to a line perpendicular to a line denoting the articular margin, as described by Court-Brown et al. [56]. Adequate neck-shaft reduction will be classified as a neck-shaft angle between 120 and 140 degrees. Angles below 120 and above 140 degrees will be classified as varus and valgus malunions, respectively. The plate position will be referenced with regard to the bicipital groove and the tip of the greater tuberosity on CT scan. Proper plate position will be classified as posterior to the bicipital groove and 5–8 mm below the tip of the greater tuberosity. The proximal humerus neck-shaft angle, head alignment, fracture reduction, and the hardware position will be recorded on standardized forms. We do not expect further imaging will be required.

#### Other radiographic outcomes

Plain x-rays evaluated by an MSK radiologist will be used to determine the presence of non-union, malunion, fracture alignment, joint arthrosis and osteopenia. For the purposes of our study, nonunions will be defined as fractures that have not healed in 3 months or those with non-progressive callus formation on 3 consecutive monthly xrays [57–61]. The pre-operative films will be used to measure baseline cortical thickness of the proximal humerus on the AP shoulder view and will allow estimation of osteopenia as described by Mather et al. [62] and a baseline and final osteoarthritis grade will be determined by the method of Weinstein et al. [63].

Complications including infection, nerve injury, bleeding, intra-articular screw penetration (hardware failure), reoperation rates and hospital re-admission rates will be assessed and recorded at all follow-up visits.

### Compliance and loss of follow-up

Crossovers are expected in a clinical trial of this nature. If crossovers occur, patients will be analyzed in the in the group to which they were initially assigned in keeping with the intention-to-treat principle.

We will take the following measures to aid in completion of follow-up: Patients will normally reside within 90 min travelling time of their surgical center. Patients are called by the research assistant two to four days before their appointment and those who do not attend their appointment will be rescheduled. If the patient misses their re-booking then the surgeon will phone the patient to encourage their attendance. In the event that a patient chooses not to return for a follow-up, the questionnaires are mailed out with a stamped return envelope.

### Statistical analysis

Patient characteristics will be summarized with descriptive statistics. Comparative analyses will be based on the full trial cohort, using the intention-to-treat (ITT) principle (i.e., based on the participant’s randomized allocation). Reasons for missing data will be examined, and appropriate imputation methods will be used to address missing follow-up data, retaining the entire cohort for the ITT analysis. Interim analyses will occur for primary outcome measure when 50% of recruited patients finish their 1-year follow-up. Using the O’Brien-Fleming criteria for sequential tests, the significance level will be 0.005 for the interim analysis and 0.049 for the final analysis. The O’Brien-Fleming boundaries, as well as clinical judgment concerning adverse events, will be used as guidelines for stopping the study early. However, this interim analysis will only occur if randomization is not yet complete.

### Primary objective analysis

A. The analysis involves a comparison of the mean Constant scores between the two groups on an intention-to-treat basis. An ANOVA will be used to assess whether there is a statistically significant difference between treatment groups for the mean Constant scores at 2 years, with stratification factor included. A non-parametric Kruskal-Wallis will be instead used if parametric tests are not justified. In addition, a Generalized Estimated Equations (GEE) analysis will be conducted to determine whether there is an effect over time (repeated measures) (i.e. pre-operatively, 3, 6, 12 and 24 months follow-ups).

### Secondary objective analysis

A. ANOVAs will be used to compare the following two-year outcome measures between the two groups: ASES, EQ-5D-5 L, PROMIS and IPAQ. In addition, the incidence proportion of complications will be derived for each group, and a comparison of the two groups will be conducted for re-operation, infection, nerve injury, hematoma, hospital readmission, and intra-articular screw penetration using a chi-square analysis. Logistic regression modeling will be used to account for variables demonstrating group imbalance.
B. The incidence of malunion, non-union, and osteoarthritis will be compared between groups using chi-square statistics (and odds ratios). Time to union will be determined with Kaplan-Meier curves with a log rank test for group comparisons.
C. The degree of association between the degree of displacement and angulation of the fracture fragments and the Constant score will be determined. Multivariable regression analysis will be carried out to determine if an association exists between fracture angle and the degree of displacement (independent variables), and functional outcomes using the Constant score (dependent variable). If such an association is identified, area under the curve plots will be used to determine at what degree of angulation or displacement a particular treatment is indicated.
D. The degree of association between the quality of surgical reduction [degree of displacement/angulation] of the fragments based on post-operative CT scan and the Constant functional outcome measure will be determined in the surgical group using a multivariable regression analysis.

### Sub-analysis

In addition to the primary and secondary analyses outlined above, several other analyses are planned, though the study is not primarily powered for these statistical analyses. We will perform a subgroup analysis on the above outcome measures to assess whether there is a difference between 3- and 4- part proximal humerus fractures. This will primarily include a subgroup analysis of the Constant score, within groups of fracture type: 3- and 4- part fractures. The second will be a multivariable assessment of the progression of osteoarthritis between pre- and post-operative shoulders. A multivariable regression analysis will also be conducted of possible factors associated with progression of shoulder osteoarthritis, including demographic variables and fracture type. The third will be a multivariable regression analysis to determine which factors may be associated with functional outcome: with the Constant score as the dependent variable, demographic and radiographic factors (including osteoporosis) will be analyzed to determine if prognostic factors exist that may assist in planning treatment.

## Discussion

The optimal management of proximal humeral fractures remains controversial [4]. Three previous randomized studies have not demonstrated a difference in patient-centered outcomes between non-operative treatment and open reduction internal fixation [44, 45]. Two of these studies, however, had relatively small numbers of patients. Surgical complications and mortality may occur with increased frequency in the elderly [42] and as such, avoidance of unnecessary surgery would decrease patient morbidity and decrease cost to the health care system.

The PROFHER trial [39] is currently the largest multi-centered randomized controlled trial to compare operative vs nonoperative treatment of proximal humerus fractures. The trial did not show a significant difference in functional outcome between treatment groups using the Oxford and SF-12 functional outcome scoring. The PROFHER trial had a few methodological limitations that the authors of the current study have sought to address. The PROFHER trial included patients from age > 16 years. The functional demands in younger patients differ and are not necessarily generalizable to older patients. Although there was a subgroup analysis for age < 65 and > 65, having wide age inclusion criteria makes interpretation of the results difficult. The current trial inclusion criteria limits the study to patients over the age > 60. Other limitations in the PROPHER study included lack of blinding, the inclusion of hemiarthroplasty in the surgical arm, lack of standardization between rehabilitation programs in the operative and nonoperative arms, and very low enrollment in certain centers; all of these potential shortcomings have been addressed in the current trial including blinding of research personnel, limiting surgical treatment to ORIF, standardization of rehabilitation protocols, and limiting enrollment to a large, high-volume, tertiary care center.

Further potential strengths include the use of pre-operative CT scan for analysis of fracture displacement/angulation, and inclusion of the IPAQ score, which is tailored towards the elderly population in order to assess the functional demands of the population within the study and how this impacts performance on the other functional assessment tools.

Our data will provide level 1 evidence that will inform the management of proximal humerus fractures in the elderly population which in turn will allow surgeons to recommend the most effective treatment for patients with this injury while taking imaging characteristics into account.

## Abbreviations

ANOVA: Analysis of Variance
ASES: American Shoulder and Elbow Society
CT: Computed Tomography
DASH: Disability Arms Shoulder and Hand
GEE: Generalized Estimated Equations
HRQol: Health Related Quality of Life
IPAQ: International Physical Activity Questionnaire
ITT: Intention to Treat
IV: Intravenous
ORIF: Open Reduction and Internal Fixation
OTA: Orthopaedic Trauma Association
PROFHER: Proximal Fracture of the Humerus Evaluation by Randomization
PROMIS: Patient-Reported Outcome Measurement Information System
QUALY: Quality Adjusted Life Years
RCT: Randomized Controlled Trial
SF-12: Short Form
